# Effects of the pandemic on the mental health of children and adolescents. review and current scientific evidence of the SARS-COV2 pandemic

**DOI:** 10.1192/j.eurpsy.2021.597

**Published:** 2021-08-13

**Authors:** P. Del Sol Calderon, A. Izquierdo, M. García Moreno

**Affiliations:** Psychiatry, Hospital Universitario Puerta de Hierro, Majadahonda, Spain

**Keywords:** COVID19, mental health, chind and adolescent

## Abstract

**Introduction:**

The coronavirus crisis has had an impact on the mental health of children and adolescents

**Objectives:**

Determine how it has affected this population and what mental pathologies are occurring

**Methods:**

Literature bibliographic review

**Results:**

School closures and lockdown have been seen to have produced higher levels of anxiety, anger, and sleep and appetite disruption. On the one hand, the children had more anxiety and regressive behaviors, and the adolescents had more isolation, depressive symptoms and even autolytic ideation. The economic crisis and lockdown have affected the family environment, having reported greater situations of domestic violence and substance use among parents. Studies show a prevalence up to 28-34% of post-traumatic stress symptoms among adolescents. In Spain it has been determined that ¼ children present anxiety and / or depression. Their parents noticed in them greater irritability, less concentration and greater feelings of loneliness There has been an increase in addiction to new technologies. This is partly a method of maintaining social relationships, but prolonged use is associated with higher levels of anxiety and depression. Regarding to patients with mental pathology, they have presented greater tantrums, especially ADHD and ASD, due to the loss of structure and routine.

**Conclusions:**

Confinement and fear of COVID have affected mental health of children and adolescents, with anxiety and depression occurring more frequently. Is highlighted the presence of feelings of loneliness among adolescents and the increase in the use of screens. Finally close to 80% of patients who had mental health conditions referred that this crisis had worsed their symtponms

